# The predictive and incremental validity of ADHD beyond the VRAG-R in a high-risk sample of young offenders

**DOI:** 10.1007/s00406-021-01352-x

**Published:** 2021-12-03

**Authors:** Priscilla Gregório Hertz, Marcus Müller, Steffen Barra, Daniel Turner, Martin Rettenberger, Wolfgang Retz

**Affiliations:** 1 Department of Psychiatry and Psychotherapy, University Medical Center of the Johannes Gutenberg-University Mainz, Untere Zahlbacher Straße 8, 55131 Mainz, Germany; 2 Institute for Forensic Psychology and Psychiatry, University Hospital, Homburg, Saarland Germany; 3 Centre for Criminology, Kriminologische Zentralstelle-KrimZ, Wiesbaden, Germany; 4 Department of Psychology, Johannes Gutenberg University of Mainz, Mainz, Germany

**Keywords:** Recidivism risk, VRAG-R, Risk assessment, Young offenders, Attention deficit/hyperactivity disorder, ADHD

## Abstract

The VRAG-R is a well-established actuarial risk-assessment instrument, which was originally developed for assessing violent recidivism risk in adult male offenders. Whether or not the VRAG-R can also predict violent recidivism in young offenders is unclear so far. In the emergence of juvenile offending, attention-deficit/hyperactivity disorder (ADHD) seems to be of major importance suggesting that it could be relevant for risk assessment as well. Thus, we examined the predictive accuracy of the VRAG-R in a high-risk sample of *N* = 106 (*M* = 18.3 years, SD = 1.8) young offenders and assessed the incremental predictive validity of ADHD symptomatology beyond the VRAG-R. Within a mean follow-up time of *M* = 13 years (SD = 1.2), *n* = 65 (62.5%) young offenders recidivated with a violent offense. We found large effect sizes for the prediction of violent and general recidivism and re-incarcerations using the VRAG-R sum scores. Current ADHD symptomatology added incremental predictive validity beyond the VRAG-R sum scores concerning the prediction of general recidivism but not of violent recidivism. The results supported the use of the VRAG-R for predicting violent recidivism in young offenders. Because ADHD symptomatology improves the predictive performance of the VRAG-R regarding general recidivism, we argue that addressing ADHD symptoms more intensively in the juvenile justice system is of particular importance concerning a successful long-term risk management in adolescents and young adults.

## Introduction

The Violence Risk Appraisal Guide-Revised (VRAG-R) [43] is a second-generation risk-assessment instrument developed to replace its predecessors, the Violence Risk Appraisal Guide (VRAG) [20] and the Sexual Offender Risk Appraisal Guide (SORAG) [33, 34]. The VRAG-R is easy to score and should work equally well concerning the assessment of recidivism risk for violent offending in any kind of adult male offender who has committed a “serious antisocial act” [21]. First cross-validation studies have shown that the VRAG-R can predict violent recidivism in different adult offender samples with moderate to large effect sizes, including violent and sexual offenders as well as offenders not criminally responsible on account of mental disorder [12, 15, 29, 53]. However, the VRAG-R, just like most other risk-assessment instruments, was primarily developed for adults and it is not yet clear, whether it can be validly used in young offenders as well.

Nonetheless, an increased use of risk-assessment instruments could be observed in the juvenile justice system in the last decades, with studies showing a growth from 33% to over 86% since the 1990s [14, 30]. Correspondingly, the number of validation studies concerning different risk assessment instruments with adolescents and young adult offender samples has as well increased during the last 2 decades [46]. In a meta-analysis, the average predictive accuracy of risk-assessment instruments for adolescent and young adult offenders based on 28 different tools yielded a moderate effect size (AUC = 0.64) [46]. In a further review including 19 studies, the predictive accuracy of six well-known risk-assessment instruments in juvenile sexual offenders (JSO) was examined [22]. As expected, the results obtained by the Structured Assessment of Violent Risk in Youth (SAVRY) [8] and the Hare Psychopathy Checklist: Youth Version (PCL:YV) [11] for sexual recidivism appeared to be weaker than specialized tools such as the Juvenile Sex Offender Assessment Protocol-II (J-SOAP-II) [32] or the Estimate of Risk of Adolescent Sexual Offence Recidivism (ERASOR) [54]. Although some of the instruments seemed promising for risk assessment among JSOs, none of them showed unequivocal positive results based on the studies included in the review with regard to predicting future offending among this population [22]. Furthermore, it is important to emphasize, that neither the PCL-R, the PCL-YV nor the PCL-SV were designed to be risk-assessment tools, nonetheless the PCL-R and its derivatives are widely used psychological instruments in forensic psychiatry and psychology [18] and several international studies support its predictive validity especially for general and violent recidivism (e.g., [17]). The study by Barra et al. [5] represents the first investigation using the VRAG-R in a sample of young offenders: They tested the validity of risk-assessment instruments such as the VRAG-R, the J-SOAP II and the ERASOR with regard to sexual, non-sexual-violent, and general criminal recidivism in a sample of 597 male juvenile sexual offenders. They found that the J-SOAP II was valid for predictions of sexual and non-sexual-violent recidivism and the ERASOR was best suited to predict sexual recidivism, whereas the VRAG-R showed potential strengths in predicting non-sexual violent recidivism, especially when committed above age 18 [5].

Beyond common risk factors included in risk-assessment instruments in young offenders, such as offending history, substance abuse, family problems, peer delinquency, and school-related problems (e.g., [23, 25, 47, 52]), externalizing psychiatric impairments have also been associated with juvenile delinquency in general and with an increased risk for criminal recidivism [1, 2, 26, 49, 51]. There is growing evidence that attention-deficit/hyperactivity disorder (ADHD) is of special importance concerning delinquency in adolescents and young adults as along with associated constructs such as intermittent explosive disorder (IED), oppositional defiant disorder (ODD), and conduct disorder (CD) [7, 39].

ADHD is a neurodevelopmental disorder beginning in childhood. Its symptoms concern interferences in executive functioning, such as attention problems, hyperactive behavior, and impulse control deficits. The association between ADHD and criminality is strongly supported [4, 48, 56]. In a systematic review and meta-analysis, prevalence rates of adult ADHD were found to be between 26 and 30% and between 35 and 47% for retrospectively assessed ADHD in childhood among adult offenders [4]. In comparison, the worldwide pooled prevalence of ADHD in children and adolescents was 5.3%, pointing out the significance of ADHD in offender populations [31]. Furthermore, individuals with ADHD are not only more likely to engage in criminal behaviors but are also younger at first conviction [55] and at first arrest [10] and were found to reoffend sooner than offenders with no ADHD [13]. A systematic review on long-term outcome studies including nine samples with a total of *N* = 15,442 children and adolescents could show that childhood ADHD was significantly associated with adolescent and adulthood arrests, convictions, and incarcerations [28]. Furthermore, individuals with ADHD were younger at onset of antisocial involvement and held an increased risk of criminal recidivism [28]. A more recent study with a considerably large follow-up of 15 years showed similar results, former adolescent and young adult offenders with ADHD reoffended 2.5 times faster and had a higher rate of recidivism and further incarcerations compared to non-ADHD participants, even when controlling for general risk factors such as antisocial personality disorder [35]. Despite the substantial long-term risk associated with ADHD for later antisocial involvement, its role in predicting further re-offending can still be considered controversial, since it seems to modulate recidivism, yet not classify as a predictive risk factor per se (e.g., [13, 35]).

Studies concerning the VRAG-R in adolescents and young adults are still rare. Further, instruments that can be used in both sexual and non-sexual violent young offenders and, thus, cover a large number of offenses are still missing. The VRAG-R could be an exception, as it was designed for this purpose. Since the VRAG-R showed some potential in predicting recidivism in young sexual offenders—although not specifically developed for these means—(e.g., [5]), we aimed at expanding the current state of knowledge concerning the predictive accuracy of the VRAG-R in the present study, by examining a high-risk sample of adolescents and young adults offenders convicted of different kinds of offenses. We hypothesized that the VRAG-R would show significant predictive accuracy for violent and general recidivism in our sample and, therefore, would significantly discriminate between young reoffenders and non-reoffenders. Because some risk factors are more age-related than others (e.g., marital status), we did not expect significant correlations between every item and the outcome measure in the present sample. Lastly, since the VRAG-R only considers externalizing problems with regard to conduct problems, we also examined the incremental predictive validity of further externalizing psychopathology in terms of ADHD symptoms beyond the VRAG-R. Based on previous research, we expected ADHD to show significant incremental predictive validity beyond the VRAG-R risk assessments. By investigating the role of ADHD in the prediction of recidivism risk in adolescent and young adult offenders, we aimed at providing further empirical evidence for the association between ADHD and (repeated) criminal behavior and so, to reinforce the importance of ADHD as a target for early treatment and prevention.

## Method

### Data collection and sample

The baseline data used in the present study were gathered between 2001 and 2002 at the Ottweiler Juvenile Detention Center in Saarland, Germany (see [35, 40, 44] for previous studies). In Germany, arrest cannot legally occur until a person is 14 years old, and juvenile law is usually applied to individuals up to 18- to 21-years-old. Juvenile sentences and pre-trial detention of male adolescents and young adults in Saarland are carried out in the Ottweiler prison. The enforcement plan of the state intends that pre-trial detention on male offenders who were under 21 years of age at the time of the offense will be consummate there. The ethics committee of the medical chamber of Saarland, Germany, had approved the study.

At the time of collecting the baseline data, out of the *N* = 170 former inmates who were initially asked to participate in the former study, *n* = 41 (24.12%) refused to sign the informed consent form or had insufficient knowledge of the German language. For individuals under the age of 18, informed consent was provided by parents or legal guardians. A total of *n* = 129 young offenders were finally included in the study. A team of psychiatrists assessed the following information during personal visits at the juvenile detention center using standardized tests, incarceration files and personal interviews: sociodemographic and biographical information, information related to the index offense, relevant psychiatric and psychological data, including clinical diagnoses, in particular antisocial personality disorder, and ADHD.

The offense the young offenders were incarcerated for at the time of baseline data collection (2001/2002) was defined as the index offense. Data concerning recidivism originated from criminal records over 15 years following their subsequent release. In Germany, criminal records encompass convictions only and do not provide information about criminal charges. Of the initial cohort, *n* = 21 were not included in the follow-up, as no criminal records were available. Two more participants were excluded in associating the data sets (1 died, 1 could not be assigned). In total, full information including criminal records for *n* = 106 young men was obtained.

On average, the *n* = 106 male young offenders were *M* = 18.33 years old at the time of index offense (SD = 1.77, 14–23). Regarding the educational level, 17% had no graduation (*n* = 18), 35.8% had an auxiliary school graduation (*n* = 38), 44.3% had a secondary school graduation (*n* = 47), and 2.8% had a high school diploma (*n* = 3). Out of the participants included in the final analyses, 68.9% (*n* = 73) had a substance related problem (dependency or abuse), 69.8% (*n* = 74) fulfilled the ADHD criteria in the childhood and 12.3% (*n* = 13) met the ICD-10 criteria for current ADHD.

Regarding index offenses, 35.8% committed a property related offense (*n* = 38), 28.3% bodily harm (*n* = 30), 11.3% narcotics related offense (*n* = 12), 4.5% homicide (*n* = 4), 3.8% sexual offenses (*n* = 2), and 0.9% arson (*n* = 1). The average arrest/detention period was *M* = 92.6 weeks (*SD* = 70.8). Furthermore, 73.6% (*n* = 78) of the young offender sample had already been convicted for a previous crime. Prior to the index offense, 46.2% (*n* = 49) of the young offenders had not been arrested and 31.1% (*n* = 33) had not been incarcerated.

### Measures and procedure

Demographic, biographic, criminological, and clinical data were collected from comprehensive incarceration files and interviews as described above. Recidivism risk was retrospectively assessed for the total sample by two independent raters using the German version of the VRAG-R [15, 37, 38]. The VRAG-R [15, 43] is an actuarial risk-assessment instrument developed to assess the risk of violent recidivism and consists of 12 predominantly static items. Its outcome measure, violent recidivism, includes any violent offenses and sexual offenses involving physical contact with the victim (sexual contact offenses, [21]. The total score can range from −27 to 51 and can be divided into nine risk bins, which are assigned to empirically derived violent recidivism estimates. The VRAG-R has shown an excellent interrater reliability with an intraclass correlation coefficient (ICC) of 0.98 (single rater, absolute agreement, [43]. In the first German cross-validation study [15] the VRAG-R also yielded an excellent interrater reliability of ICC = 0.97 (*p* < 0.001; ICC[A,1]; random effects, single measure, absolute agreement; [27]).

In assessing recidivism risk in our sample, missing VRAG-R-items were prorated (for details see [34]. No additional informed consent was necessary when evaluating the collected baseline data for the VRAG-R assessments.

For the present study, VRAG-R assessments were operationalized using its sum scores as independent variable. Data concerning re-offenses originated from criminal records for a maximum period of 15 years after release (*M* = 13, SD = 1.2) and corresponded to new convictions. The outcome variable recidivism was operationalized as: 1. General recidivism, i.e., any new reconviction regardless of offense type, 2. Violent recidivism, which includes any violent as well as contact sexual offenses, and 3. Re-incarcerations, which is a more conservative measure (charges < convictions < incarceration).

Furthermore, diagnosing ADHD in juveniles and adults requires the retrospective assessment of symptoms in childhood. Therefore, in the present study, ADHD symptoms in childhood were assessed using the German short version of the Wender Utah Rating Scale (WURS-k; [45], org. [50]. The WURS-k comprises 25 items for the retrospective assessment of ADHD in childhood with a sensitivity of 85% and a specificity of 76% at a cutoff of 30 points. Furthermore, the WURS-k yielded excellent internal consistency (*α* = 0.91) and an excellent split-half correlation of *r* = 0.85 [41]. The presence of current ADHD symptoms was assessed using the ADHD-SB questionnaire (ADHD-SB,[45], which is an instrument based on the ICD-10 research criteria and the diagnostic criteria according to DSM-IV. Since relevant information about the severity of the psychiatric impairments goes missing using categorical representations and because previous studies claimed subclinical ADHS should also be included in research [35], we chose a dimensional approach and applied the sum scores of both WURS-k and ADHD-SB to calculate the incremental predictive validity of the ADHD symptomology beyond the VRAG-R risk assessments (e.g., [6].

### Statistical analyses

First, we examined the interrater reliability of the VRAG-R using an intraclass correlation coefficient (ICC) by comparing the results of two independent trained raters (PGH, MM) for each subject in the sample. Both raters were blind to each other’s ratings and to the outcome measures.

Second, we investigated the discriminability of the VRAG-R between recidivist and non-recidivist young offenders by calculating the area under the curve (AUC). Since AUC values are not sensitive to base rate effects [16, 42], they allow comparisons between different scales and independent studies [19]. Referring to Cohen [9], Rice and Harris [42] proposed the following interpretation criteria for AUC-values in terms of effect sizes: AUC ≥ 0.72 can be classified as “large”, AUC = 0.64-0.71 as “moderate”, and significant AUC ≤ 0.63 as “small.” Furthermore, point biserial correlations between every item and the outcome measure violent recidivism were calculated.

At last, stepwise Cox regression analyses were used to examine the incremental predictive validity of childhood and current ADHD related symptoms beyond the VRAG-R assessments. Cox regression is based on survival analysis and relates predictor variables with the survival time until an event (i.e., reoffense) happens. The effect of the independent variable is expressed by hazard ratios (exp[B] or HRs) which is a measure for the relationship between the probabilities of two groups. In the present article Cox regression analyses were utilized in two ways: First, to conduct a time-independent examination of the predictive accuracy of the VRAG-R expressed by hazard ratios (exp[B] or HR)—a hazard ratio of 1.10 for example indicates that each one-score increase on the scale increases the hazard by a factor of 1.10, or 10%. Second, Cox regression models were used to indicate the incremental predictive validity of impairments caused by ADHD beyond the VRAG-R risk assessments. For the latter, sequential Cox regression models are generally regarded as an appropriate method (e.g., [24], since they provide the Wald statistic that, if significant, indicates that the scale adds incremental validity to the other scale(s) included in the model [3].

Therefore, VRAG-R sum scores were included in the first step and the dimensionally measured childhood and current ADHD symptoms based on the WURS-k and the ADHS-SB, respectively, in the second step of the Cox regression analyses for both general and violent recidivism. All statistical analyses were performed using SPSS 26 (IBM).

## Results

### Interrater reliability of the VRAG-R

**Fig. 1:**
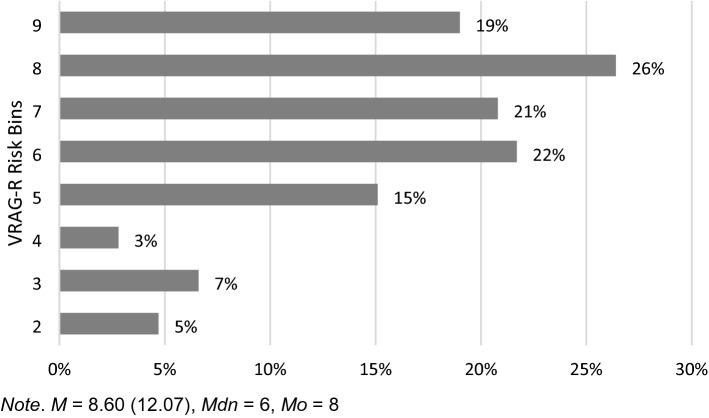
Sample distribution over the VRAG-R risk bins (*N* = 106)

The interrater reliability for the VRAG-R total score was found to be good (ICC = 0.811, *p* < 0.001; 95% CI 0.735–0.867, ICC[A,1]; [27]). The mean VRAG-R sum score in our sample was *M* = 8.60 (SD = 12.07, Min = −22, Max = 27 points), whereas the median of the risk categories was Mdn = 6 and the mode Mo = 8. For a detailed distribution of VRAG-R ratings in our sample, see Fig. 1. The mean follow-up time after release from juvenile detention was *M* = 13.1 (SD = 1.2), the average age of the individuals at date of retrieval of the Federal Central Register was *M* = 33.7 (SD = 2.1).

### Predictive validity

The recidivism rates in our sample were 84% (*n* = 89) for general recidivism and 61.3% (*n* = 65) for violent recidivism. In total, 75.5% (*n* = 80) of the young offenders were re-incarcerated after release.

VRAG-R sum scores yielded large, significant effect sizes concerning the prediction of violent (AUC = 0.733) and general (AUC = 0.861) recidivism as well as for re-incarceration (AUC = 0.874, see Table 1). Furthermore, six out of the twelve VRAG-R items showed significant positive correlations with violent recidivism as defined for the VRAG-R (Item 1—Living with both biological parents, Item 2—Elementary school maladjustment, Item 6—Failure on conditional release, Item 9—Admissions to correction, Item 10—Conduct disorder, Item 12—Antisociality). For further results and comparisons with correlation coefficients with the developmental study, see Table 2.

**Table 1:** Predictive validity of the VRAG-R sum scores for violent and general recidivism and for further re-incarcerations (*n* = 106)

	Violent		General	Re-incarceration
AUC (95% CI)				
Recidivists	*n* = 65	*n* = 89		*n* = 80
VRAG-R sum scores	0.733*** (0.636–0.829)	0.861*** (0.775–0.946)		0.874*** (0.805–0.943)

*AUC *area under curve

***p < 0.001

**Table 2:** Correlations between VRAG-R items and the violent recidivism^a^ in the present study and in the development study (*N* = 106)

VRAG-R items	Score range	*r*^*b*^ (present study)	*r*^*b*^ [43]
1. Lived with both biological parents	4	0.37***	0.18**
2. Elementary school maladjustment	7	0.24**	0.30***
3. Substance abuse	6	0.10	0.22*
4. Marital status at time of index offense	2	0.12	0.12*
5. Non-violent criminal history index offense	8	0.07	0.29***
6. Failure on conditional release	6	0.35***	0.30***
7. Age at index offense	9	–	0.27***
8. Violent criminal history	6	0.11	0.24***
9. Admissions to corrections	8	0.33***	0.31***
10. Conduct disorder	7	0.21*	0.30***
11. Sex offending history	5	0.05	0.19***
12. Antisociality	12	0.23**	0.37***

Differently from Rice et al. [43] we had used new convictions, not charges

*r*^*b*^ point biserial correlation

^a^Using variable follow-up time periods

***p < 0.001, **p < 0.01, *p < 0.05 (two-tailed)

### Incremental predictive validity

Relevant results of the sequential Cox regression analysis are presented in Table 3. Current ADHD symptomology showed statistically significant incremental predictive validity beyond VRAG-R assessments regarding general recidivism (*Wald* (1) = 3.896, *p* = 0.048, *Exp (b)* = 1.023, 95% CI = [1.000–1.047]). No significant incremental predictive validity was found regarding violent recidivism and re-incarcerations. Childhood ADHD-symptoms did not contribute to the VRAG-R risk assessments regarding the prediction of any kind of recidivism (see “Appendix” for all results).

**Table 3:** Incremental predictive validity of current ADHD symptoms beyond the VRAG-R assessments for the prediction of general recidivism (*N* = 106)

	χ^2^				Regression coefficient			RR	
	*χ*^2^	*df*	*p*	*b*	SE	Wald	*p*	Exp(B)	95% CI
Step 1									
VRAG-R sum scores	15.133	1	< 0.001	0.050	0.014	13.287	< 0.001	1.052	1.024–1.081
Step 2									
VRAG-R sum scores				0.043	0.014	8.953	0.003	1.044	1.015–1.074
Current ADHD	3.655	1	0.056	0.023	0.012	3.896	0.048	1.023	1.000–1.047

## Discussion

In the present study, we retrospectively examined the predictive validity of the VRAG-R in a sample of adolescent and young adult offenders within a mean follow-up period of 13 years. Furthermore, we investigated the incremental predictive validity of ADHD symptomatology beyond the VRAG-R. To our knowledge, evidence of the validity of the VRAG-R in juvenile and young adult offenders has been limited so far and our study represents the first investigation of the feasibility of the VRAG-R in a sample of general young offenders. Moreover, our investigation is among the first studies that have examined the predictive incremental validity of ADHD symptomatology with regard to re-offending beyond the effects of specific risk-assessment instruments.

Our results indicated a good predictive accuracy with a large effect size of the VRAG-R concerning violent recidivism in juvenile and young adult offenders, comparable to the one found in previous research in samples of adult violent and sexual offenders [15, 43] and juvenile sexual offenders [5]. Whereas in the latter study only juveniles convicted for sexual offenses were included, we recruited a rather diverse sample of violent and nonviolent adolescent and young adult offenders. Thus, there is accumulating scientific evidence that the VRAG-R can be used validly to assess violent recidivism risk not only in adult offenders—the population it was initially developed for—but also in juvenile offenders who committed different types of offenses. Additionally, the VRAG-R reliably predicted violent recidivism in juvenile and young adult offenders across a mean follow-up interval as long as 13 years. All these findings account for recidivism as defined by re-offenses as well as re-incarcerations.

Nevertheless, when comparing correlation coefficients between single items and violent recidivism in adult and juvenile samples, some considerable differences were found which should be regarded when using the VRAG-R in younger offenders or when trying to optimize the item composition in future research approaches. Whereas in both the development study and in other cross-validation studies with adult offenders every item correlated significantly with violent recidivism (e.g., [15, 43]), only 6 out of the 12 items showed statistically significant effect sizes in the current sample. The items that did not correlate with violent recidivism were the following: Substance use, marital status at time of index offense, nonviolent criminal history before index offense, age at index offense, violent criminal history, and sex offending history. For most items, it is conceivable that the missing correlation with violent recidivism is due to the lower age of our participants compared to those the VRAG-R was initially developed for. For example, it is relatively unlikely that an offender at age 18 or younger has been married at the time of the index offense, thus, it is obvious that this item is much less relevant for risk assessment in juvenile offenders than in adult offenders. On the other hand, the item “lived with both biological parents” showed a much stronger association with violent offending in our juvenile and young adult offenders than in the development sample of adult offenders [43]. This suggests that close bondings are indeed just as important in young offenders, or even more relevant, than in adult offenders, since the meaning of certain attachment figures changes with increasing age. Possibly, an item asking for close peer-relations rather than marital status could be more appropriate for young offenders. The same accounts for age at index offense, which could not be meaningfully scored within the present sample due to the young age of our participants with all participants falling into the age category “26 years or younger”. Thus, in young offenders, age categories would have to be adjusted.

Interestingly, variables pointing towards an antisocial lifestyle, like substance abuse problems, for instance having alcohol and drug problems before the age of 18 and involved in the offending history, as well as the nonviolent and violent criminal history, were also not significantly correlated with violent recidivism in our sample. The prevalence of substance abuse-related problems in our sample is extremely high, so there is little variance, and the item showed no significant correlation with the outcome. Similarly, the individuals in our sample had criminal records indicating a high prevalence of convictions due to previous crimes. Generally, juveniles have fewer entries criminal records than adult offenders, not only because they had less time to offend before the index offense, but also because the threshold for convicting a juvenile in the German legal system is considerably high. Nevertheless, the correlation between conduct disorder (Item 10) and violent recidivism indicates that behavior pointing towards an antisocial lifestyle is also important in juvenile offenders. These findings suggest that in adolescent and young adult offenders a lower threshold than in adults could make sense to capture an antisocial lifestyle as a risk factor for violent recidivism. To solve this problem, it could be considered to include other forms of rule violations over and above previous convictions. Notably, item 12 (antisociality), which is to be retrieved from facet 4 of the PCL-R correlated considerably with violent recidivism in our sample. Although the PCL-R should only be administered in individuals over 18 years old, this result emphasizes once again that an antisocial personality and lifestyle is a risk factor of outstanding relevance not only in adults but also in adolescent and young adults. Overall, we conclude that the VRAG-R can also be used in juvenile and young offenders; however, after the analyses on item level, the results indicated some starting points for future research aiming to look for potential improvement by adapting some of the VRAG-R items for this offender group.

Previous research has shown that ADHD is a relevant risk factor for recidivism, especially among juvenile offenders. It could also be shown that offenders with ADHD miss more appointments with their probation service, show more problems during the placement, etc. (e.g., [48]). In the present study, ADHD symptoms showed a small significant incremental predictive validity regarding general recidivism, even though there is considerable overlap between some risk factors included in the VRAG-R, such as elementary school maladjustment and conduct disorder, and impairments caused by ADHD symptomology. However, the small effect size regarding the incremental predictive validity of ADHD beyond the VRAG-R assessments fits findings of previous studies insofar as it does not seem to be ADHD alone which predicts recidivism, but rather the junctions of ADHD and conduct problems (e.g., [39]) and the association of ADHD and further externalizing psychopathology like emotion regulation or intermittent explosive disorder (e.g., [7]).

Nevertheless, there are limitations to be accounted for in our study. First, we examined a small, high-risk sample of male detainees, so that our results are just partially transferable to all juvenile and young adult offenders. Furthermore, the VRAG-R assessments were made retrospectively, resulting in some missing information. Even though we applied the officially recommended prorating procedure [21, 37, 38], a prospective study design would provide a better way for validating risk-assessment instruments for their clinical and forensic use. Yet, strengths of the present study include the detailed diagnostic assessment the adolescent and young adult offenders went through, the considerably large follow-up interval, and the dimensional perspective on ADHD symptomatology.

For future studies, the development of new age categories with more variance in young offenders could be a promising starting point, to increase the predictive relevance of the age-related risk factors. Other items could also be adjusted for this age range, for instance, close peer-relations could substitute marital status and a lower threshold to capture an antisociality related lifestyle as a risk factor for violent recidivism could be discussed. Furthermore, instead of the PCL-R, the PCL-YV should be used, to examine the developmental characteristics of comparatively young offenders more appropriately. Further, besides using a dimensional approach on the symptomology of ADHD, it is also important to consider ADHD subtypes in future research (e.g., [36]), which could be implemented using for instance the clinician-rated scale *Wender–Reimherr Adult Attention Deficit Disorder Scale* (WRAADDS) [7]. Conclusively, using a dimensional representation of ADHD and including ADHD subtypes in research could bring relevant contributions to the legal prognosis.

Finally, the VRAG-R is valid for the use in adolescent and young adult offenders under certain conditions. However, adaptations for a standard use in young offender populations are still necessary and should be further evaluated in future research. In addition, our results provide evidence that considering ADHD symptoms could further increase predictive accuracy when assessing the risk of criminal recidivism among adolescent and young adult offenders**.** Since ADHD is a treatable condition and has such a high prevalence among young offenders, it should not only be considered in risk assessment, but also in risk management [39].

## Data Availability

Not applicable.

## Appendix

See Tables 4, 5, 6, 7 and 8.

**Table 4:** Incremental predictive validity of childhood-ADHD symptoms beyond the VRAG-R assessments for the prediction of general recidivism (*N *= 106)

	χ^2^				Regression coefficient			RR	
	*χ* ^2^	*df*	*p*	*b*	SE	Wald	*p*	Exp(B)	95% CI
Step 1									
VRAG-R sum scores	15.133	1	< 0.001	0.050	0.014	13.287	< 0.001	1.052	1.024–1.081
Step 2									
VRAG-R sum scores				0.043	0.014	8.953	0.003	1.044	1.015–1.074
Childhood ADHD	0.931	1	0.335	0.011	0.011	0.932	0.334	1.011	0.989–1.033

**Table 5:** Incremental predictive validity of childhood-ADHD symptoms beyond the VRAG-R assessments for the prediction of violent recidivism (*N* = 106)

	χ^2^				Regression coefficient			RR	
	*χ* ^2^	*df*	*p*	*b*	SE	Wald	*p*	Exp(B)	95% CI
Step 1									
VRAG-R sum scores	0.705	1	0.401	0.011	0.013	0.679	0.410	1.011	0.985–1.038
Step 2									
VRAG-R sum scores				0.003	0.017	0.040	0.841	1.0403	0.970–1.038
Childhood ADHD	0.472	1	0.492	0.010	0.015	0.471	0.493	1.010	0.0981–1.041

**Table 6:** Incremental predictive validity of childhood-ADHD symptoms beyond the VRAG-r assessments for the prediction of re-incarcerations (*N* = 106)

	χ^2^				Regression coefficient			RR	
	*χ* ^2^	*df*	*p*	*b*	SE	Wald	*p*	Exp(B)	95% CI
Step 1									
VRAG-R sum scores	5.979	1	0.014	0.036	0.015	5.491	0.019	1.037	1.006–1.069
Step 2									
VRAG-R sum scores				0.028	0.018	2.557	0.110	1.029	0.994–1.065
Childhood ADHD	0.752	1	0.386	0.011	0.013	0.756	0.385	1.011	0.986–1.036

**Table 7:** Incremental predictive validity of current ADHD symptoms beyond the VRAG-R assessments for the prediction of violent recidivism (*N* = 106)

	χ^2^				Regression coefficient			RR	
	*χ* ^2^	*df*	*p*	*b*	SE	Wald	*p*	Exp(B)	95% CI
Step 1									
VRAG-R sum scores	0.705	1	0.401	0.011	0.013	0.679	0.410	1.011	0.985–1.038
Step 2									
VRAG-R sum scores				0.010	0.015	0.442	0.506	1.010	0.981–1.040
Current ADHD	0.019	1	0.891	0.002	0.015	0.019	0.891	1.002	0.973–1.032

**Table 8:** Incremental predictive validity of current ADHD symptoms beyond the VRAG-R assessments for the prediction of re-incarcerations (*N* = 106)

	χ^2^				Regression coefficient			RR	
	*χ* ^2^	*df*	*p*	*b*	SE	Wald	*p*	Exp(B)	95% CI
Step 1									
VRAG-R sum scores	5.979	1	0.014	0.036	0.015	5.491	0.019	1.037	1.006–1.069
Step 2									
VRAG-R sum scores				0.032	0.016	4.115	0.043	1.033	1.001–1.066
Current ADHD	1.305	1	0.253	0.013	0.011	1.347	0.246	1.013	0.991–1.036
